# A Cross-Sectional Study of the Trends in Cardiovascular Mortality Among African Americans With Hypertension

**DOI:** 10.7759/cureus.40437

**Published:** 2023-06-14

**Authors:** Eseosa Urhoghide, Nkemputaife P Onyechi, Okelue E Okobi, Victor A Odoma, Omolola Okunromade, Adole A Moevi, Olusayo Louise-Oluwasanmi, Soji Ojo, Nkechinyere M Harry, Eyitope Awoyemi, Cherechi G Sike, Bright C Nwatamole, Joseph A Agbama, Endurance O Evbayekha

**Affiliations:** 1 Internal Medicine, Hudson Regional Hospital, Secaucus, USA; 2 Internal Medicine, University Hospitals Geauga Medical Center, Cleveland , USA; 3 Family Medicine, Medficient Health Systems, Laurel, USA; 4 Family Medicine, Lakeside Medical Center, Belle Glade, USA; 5 Cardiology/Oncology, Indiana University (IU) Health, Bloomington, USA; 6 Public Health, Jiann-Ping Hsu College of Public Health, Georgia Southern University, Savannah, USA; 7 Internal Medicine, Avalon University School of Medicine, Willemstad, CUW; 8 Internal Medicine, Howard University College of Medicine, Washington, DC, USA; 9 Psychiatry, University of Texas Health Science Center at Houston, Dallas, USA; 10 Internal Medicine, National Pirogov Memorial Medical University, Vinnytsia, UKR; 11 Family Medicine, Ekiti State University Teaching Hospital, Ado Ekiti, NGA; 12 General Practice, Windsor University School of Medicine, Cayon, KNA; 13 Cardiology, Calderdale and Huddersfield NHS Foundation Trust, Huddersfield , GBR; 14 Internal Medicine, University of Calabar Teaching Hospital, Calabar, NGA; 15 Internal Medicine, St. Luke's Hospital, Chesterfield, USA

**Keywords:** hypertension related mortality, cardiovascular-related mortality, hypertension, awareness of cardiovascular disease, african american/black

## Abstract

Background and Objective

In the United States, hypertension remains a significant cause of cardiovascular disease mortality and morbidity, affecting various racial and ethnic groups. High blood pressure is a common health concern, given its high frequency among all populations and racial groups in the United States; nevertheless, the condition remains untreated in most individuals. It affects a significant number of individuals in the African American community and contributes to a notable proportion of deaths. Arguably more prevalent, severe, and tends to occur earlier in African Americans compared to some other races. This lack of blood pressure control may contribute to the increasing mortality rates associated with hypertension-related cardiovascular diseases in the United States, while notable race and sex disparities persist. This study aims to compare the number of deaths caused by each cardiovascular disease (hypertension) in African Americans to those of people of other races.

Methodology

To understand the impact of hypertension on mortality rates among different racial groups, this study utilized the Centers for Disease Control and Prevention’s Wide-Ranging Online Data for Epidemiologic Research (CDC WONDER) dataset, which includes death certificates filed in the United States. The research focused on individuals aged 25 years or older with a mention of hypertension and cardiovascular disease as the underlying cause of death between 1999 and 2019. The study analyzed hypertension-associated deaths by different cardiovascular disease subtypes, such as ischemic heart disease, heart failure (HF), and cerebrovascular diseases that include acute ischemic attacks, which are the most frequent in the United States, with specific assessments for African Americans, White, and other races' decedents.

Results

The study findings indicated that African American males had higher mortality rates from cardiovascular diseases compared to African American females. The prevalence of hypertension was also higher among African Americans (87.47%) compared to Whites (30.33%), Asian/Pacific Islanders (40.26%), and American Indians/Alaska Natives (61.18%). Additionally, the study identified regional variations in mortality rates, with states like Arizona, California, Texas, Florida, and Washington having higher rates, while Vermont, North Dakota, and Wyoming had lower rates. The northwest region had lower mortality compared to the western and southwestern regions.

Conclusions

Within the studied period, there was an increase in the prevalence of mortality due to hypertension amongst African Americans when compared to other races. These findings underscore the pressing need to address the increasing prevalence of hypertension and mortality rates among African American. More efforts should focus on prevention of CVD and hypertension and the associated risk factors based on the World Health Organization (WHO) recommendations, which include the promotion of healthy lifestyle behaviors, improvement of access to quality healthcare, and implementation of culturally sensitive interventions tailored for African American communities.

## Introduction

Ethnic and racial disparities in cardiovascular disease (CVD) mortality have remained pervasive throughout the United States [1]. Although initial studies have indicated that adult African American men and women have an increased rate of mortality as a result of stroke, heart failure, and severe myocardial infarctions in comparison to other races and ethnic groups, gender-/sex-based disparities about cardiovascular conditions have not been aptly described at the national level [2]. African American men and women have disproportionate experiences of economic, social, and environmental limitations and barriers, and structural racism that have contributed to the increased burden of cardiovascular mortality and associated risk factors [3,4]. Exceptionally, African American women have been observed to face various challenges at the sexism and racism intersections, in addition to negative maternal outcomes resulting from CVD that has adverse effects on the women’s cardiovascular health [5].

Moreover, in 2012, the life expectancy of African Americans was 3.4 years shorter than that of Whites (75.5 versus 78.9 years, respectively). The contrasts are most striking when studied by race and sex: White women have the longest life expectancy at 81.4 years, followed by Black women at 78.4 years, White men at 76.7 years, and Black men at 72.3 years [6]. CVD remains the most common cause of morbidity and mortality among African Americans in the United States [7]. Although various targeted public health interventions have been executed, hypertension rates have remained persistently high among African Americans compared to other racial groups in the United States. In recent times, the number of deaths associated with hypertension has significantly increased in the United States, even as the marked sex-race disparities have also persisted. Nonetheless, hypertension has been rarely listed as the main underlying cause of mortality despite being acknowledged as contributing to deaths via CVDs, including heart failure, ischemic heart disease, and cerebrovascular disease.

Moreover, despite the view that the overall mortality from coronary artery disease (CAD) is decreasing in the United States, Black Americans still have higher CAD mortality than Whites and greater morbidity from CAD and related conditions, including metabolic syndrome, hypertension, diabetes, stroke, and obesity [8]. In the United States, hypertension accounts for 50% of the racial disparities in mortality between Blacks and Whites. So far, genome-wide association studies have failed to identify distinct genetic causes for the excess burden in this population. Pathophysiology is likely to be complex, involving the interaction of genetic, biological, and social factors that are prevalent among African Americans [9].

## Materials and methods

Data availability

The Centers for Disease Control and Prevention’s Wide-Ranging Online Data for Epidemiologic Research (CDC WONDER), a comprehensive online publicly available health information system and datasets, were used in this project. The results are, therefore, replicable using the methods described in this study [10].

Data source

We examined *Multiple Causes of Death* data from the CDC WONDER, which includes county-level national mortality and population data. The data is based on death certificates for US citizens. Each death certificate includes a single cause of death, up to 20 additional multiple causes, and demographic information. The number of deaths, crude death rates, and age-adjusted death rates can be obtained by location (national, state, and county in the United States), age group, race, Hispanic ethnicity, gender, year and month of death, weekday of death, place of death, autopsy status, and underlying and multiple causes of death.

The underlying cause of death is defined by the World Health Organization (WHO) as "the disease or injury which initiated the train of events leading directly to death, or the circumstances of the accident or violence which produced the fatal injury." The underlying cause of death is selected from the conditions entered by the physician on the cause-of-death section of the death certificate. When more than one cause or condition is entered by the physician, the underlying cause is determined by the sequence of conditions on the certificate, provisions of the International Classification of Diseases (ICD), and associated selection rules and modifications.

We selected cardiovascular-related deaths attributed to hypertension identified using the ICD-10 codes I10-I15 [10]. A Coroner provides information regarding ethnicity and race as provided by the deceased next of kin or by observation in the absence of an available next of kin. Ethnicity and race were determined using the death certificates reporting race and Hispanic origin separately following the standards outlined by the Office of Management and Budget [11]. This study did not require the institutional review board's approval as the analysis used government-issued public data without individually identifiable information.

The CDC provides clean and deidentified data files that are publicly accessible for analysis purposes. Throughout our analysis of this cross-sectional data, we have strictly adhered to the guidelines outlined by Strengthening the Reporting of Observational Studies in Epidemiology (STROBE). These data files do not include any personal identifiers, ensuring the privacy and confidentiality of individuals.

Data extraction

We extracted the number of cardiovascular deaths attributable to hypertension and hypertension-related diseases between 1999 and 2019. The data was abstracted based on age, ethnicity, sex, state, and county. The US Census Bureau used five racial/ethnic groups to categorize people: non-Hispanic American Indian and Alaskan Native, non-Hispanic Asian and Pacific Islander, non-Hispanic Black (or African American), non-Hispanic White, and Hispanic. Mortality data was stratified by sex and across these groups. Geographical stratification of mortality rates included rates for the 50 states, urban and rural counties, and the nine US Census divisions (New England, Middle Atlantic, East North Central, West North Central, South Atlantic, East South Central, Mountain, and Pacific). We did not look at data for the US territory. We grouped the decedents into five-year categories, starting from 18 years up to 65 years to focus on premature mortality. We identified race and ethnicity as African American/Black adults, White adults, Hispanic adults, and Asian/Pacific islanders. Counties were classified using the National Center for Health Statistics 2013 urban-rural classification scheme, which stratified counties into large, medium, metro, small metro, and rural counties.

## Results

Total trends in mortality by state

**Figure 1:**
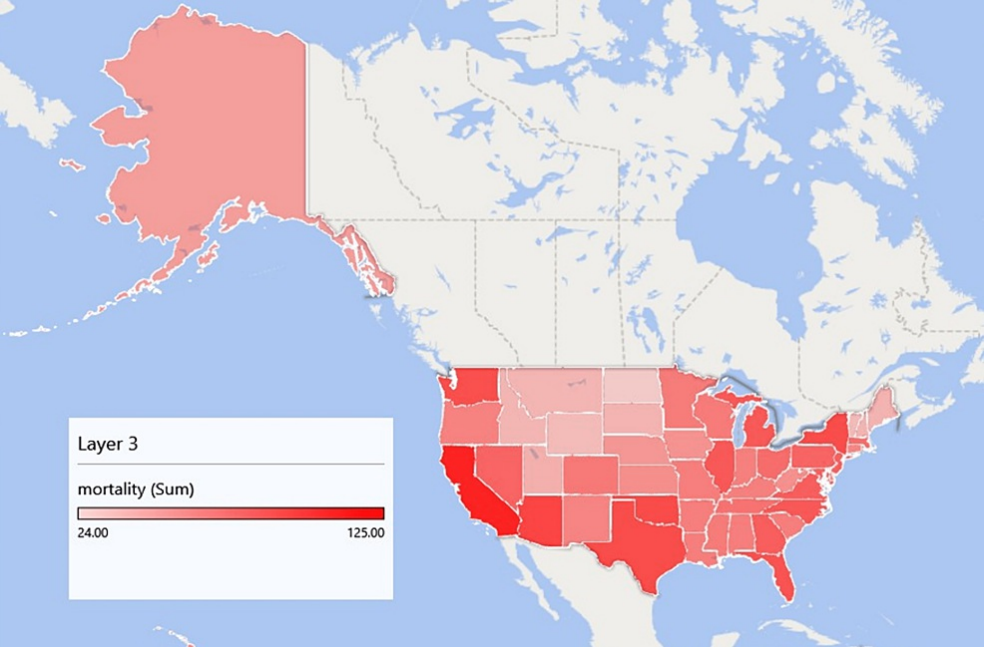
Hypertension-related cardiovascular mortality by states. Figure credit: This figure is an original creation by the authors of this manuscript as obtained from the result of this study.

Table 1 and Figure 1 illustrate cardiovascular mortality from hypertension, stratified by states. A total of 3,337 cardiovascular mortality related to hypertension was recorded. Individual state mortalities were calculated as a percentage of the total mortality; the cumulative percentage was also evaluated and recorded. The states with the highest mortality were Arizona, California, Texas, Florida, and Washington, while Vermont, North Dakota, and Wyoming had the lowest number of mortalities. Regionally, the northwest area had less mortality, with most of the mortality being concentrated in the southwest and western states.

**Table 1:** Trends in cardiovascular mortality related to hypertension by states (all races, 1999-2019).

States	Frequency	Percentage	Valid percentage	Cumulative percentage
Alabama	64	1.9	1.9	4.2
Alaska	43	1.3	1.3	5.5
Arizona	102	3.1	3.1	8.6
Arkansas	64	1.9	1.9	10.5
California	125	3.7	3.7	14.2
Colorado	67	2.0	2.0	16.2
Connecticut	57	1.7	1.7	18.0
Delaware	50	1.5	1.5	19.4
District of Columbia	52	1.6	1.6	21.0
Florida	92	2.8	2.8	23.8
Georgia	85	2.5	2.5	26.3
Hawaii	51	1.5	1.5	27.8
Idaho	28	.8	.8	28.7
Illinois	89	2.7	2.7	31.3
Indiana	63	1.9	1.9	33.2
Iowa	50	1.5	1.5	34.7
Kansas	53	1.6	1.6	36.3
Kentucky	63	1.9	1.9	38.2
Louisiana	64	1.9	1.9	40.1
Maine	29	.9	.9	41.0
Maryland	82	2.5	2.5	43.5
Massachusetts	74	2.2	2.2	45.7
Michigan	88	2.6	2.6	48.3
Minnesota	65	1.9	1.9	50.3
Mississippi	65	1.9	1.9	52.2
Missouri	64	1.9	1.9	54.1
Montana	28	.8	.8	55.0
Nebraska	48	1.4	1.4	56.4
Nevada	81	2.4	2.4	58.8
New Hampshire	28	.8	.8	59.7
New Jersey	82	2.5	2.5	62.1
New Mexico	60	1.8	1.8	63.9
New York	97	2.9	2.9	66.8
North Carolina	89	2.7	2.7	69.5
North Dakota	24	.7	.7	70.2
Ohio	75	2.2	2.2	72.5
Oklahoma	98	2.9	2.9	75.4
Oregon	61	1.8	1.8	77.2
Pennsylvania	80	2.4	2.4	79.6
Rhode Island	39	1.2	1.2	80.8
South Carolina	64	1.9	1.9	82.7
South Dakota	29	.9	.9	83.6
Tennessee	71	2.1	2.1	85.7
Texas	97	2.9	2.9	88.6
Utah	32	1.0	1.0	89.6
Vermont	25	.7	.7	90.3
Virginia	85	2.5	2.5	92.9
Washington	95	2.8	2.8	95.7
West Virginia	52	1.6	1.6	97.3
Wisconsin	66	2.0	2.0	99.3
Wyoming	25	.7	.7	100.0
Total	3337	100.0	100.0	

Trends in mortality by gender and race

Table 2 provides insights into the trends of mortality rates between males and females based on data drawn from the CDC WONDER registry. It shows that of the total recorded deaths within the observed period (3,337), 47.6% were females and 50% were males. Based on the data provided, one may conclude that males have a higher mortality rate from hypertensive-CVDs than their female counterparts. Moreover, the cumulative percentage indicates that CVD is more pronounced in males (100%) than in females in all populations.

**Table 2:** Mortality by gender and race in African Americans. NA, not applicable

Variable	Frequency	Percentage	Valid percentage	Cumulative percentage
Female	1,590	47.6	47.6	50
Male	1,670	50	50	100
Others or those who did not identify by sex	77	2.4	2.4	NA
Total	3,337	100	100	NA

Descriptive of prevalence and mortality among African Americans compared to other races with hypertension

Table 3 provides descriptive statistics on the occurrence and mortality of hypertension among different racial groups in the United States. The datasets show that African Americans have the highest mean prevalence of hypertension at 87.47% in comparison to other races/ethnicities in the United States. This is significantly higher in comparison to other racial groups. Thus, based on the findings, Whites have a mean prevalence of 30.33%, Asian/Pacific Islanders have a mean prevalence of 40.26%, and American Indians/Alaska Natives have a mean prevalence of 61.18%. Further, the standard deviations for each racial group have been included to indicate the extent of variation in hypertension prevalence within the various racial groups. The African Americans had a standard deviation of 97.52, suggesting a wide range of prevalence rates within the population. Similarly, the other racial groups have standard deviations indicating considerable hypertension prevalence variability. For instance, White Americans have a standard deviation of 41.52, Asian/Pacific Islanders have a standard deviation of 48.13, and American Indians/Alaska Natives have a standard deviation of 61.50.

**Table 3:** Mortality trends by races.

Races	N	%	Mean	SD	SE	95% CI for mean	
						Lower bound	Upper bound
American Indian or Alaska Native	144	4.74	61.18	61.50	5.13	51.05	71.31
Asian or Pacific Islander	282	11.72	40.26	48.13	2.87	34.62	45.90
Black or African American	883	36.7	87.47	97.52	3.28	81.02	93.91
White	1126	46.8	30.33	41.52	1.24	27.90	32.76

**Figure 2:**
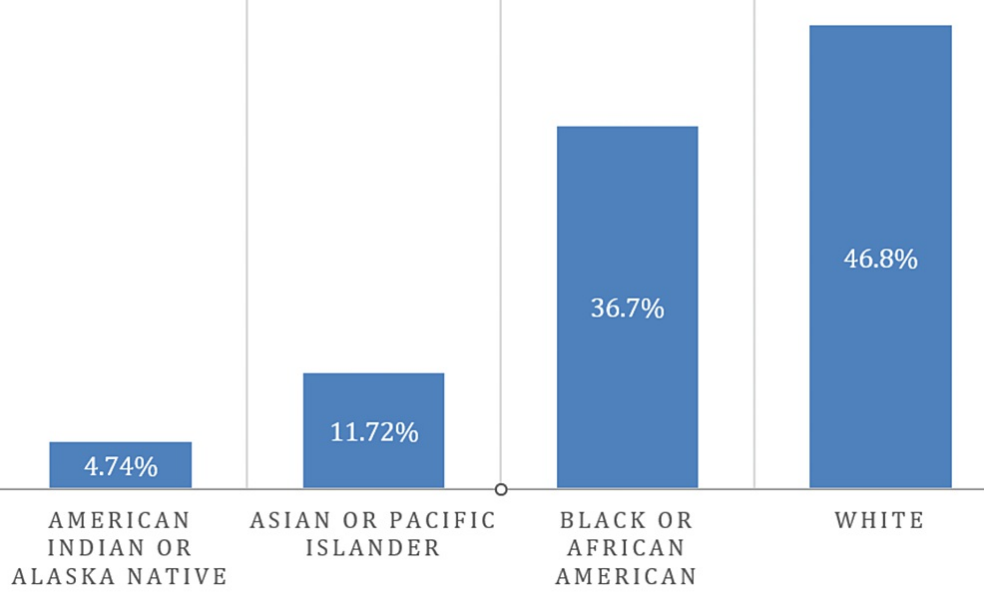
Mortality among African Americans compared to other races with hypertension.

A t-test was used in the calculation of a 95% confidence interval (CI). Thus, the 95% CI for the mean prevalence provides a range within which the true population means will likely fall. For African Americans, the 95% CI ranges from 81.02 to 93.91. This means that one can be 95% confident that the true population means the prevalence of hypertension among African Americans lies within this interval. Such findings underscore the presence of significant disparities in hypertension prevalence among different racial groups, in addition to highlighting the need to adherence to the European Society of Cardiology (ESC) or European Society of Hypertension (ESH) guidelines for targeted interventions and healthcare strategies, specifically tailored to address hypertension prevention, management, and treatment within the African American community. Also, the proportion of mortality for African Americans was 36.7%, as shown in Figure 2.

Analysis of variance (ANOVA) of prevalence and mortality among African Americans compared to other races with hypertension

Table 4 analyzes the variance/ANOVA to compare the hypertension prevalence and mortality rates among African Americans to other racial groups. As the ANOVA results indicate, the between-groups sum of squares (SS) is 1,680,536.50 with 3 degrees of freedom, resulting in a mean square (MS) of 560,178.83. This indicates the variability in prevalence and mortality rates among the racial groups. The high F-value of 118.22 suggests a significant difference between the groups regarding CVD-related outcomes. Additionally, the higher *P*-value of 0.001 confirms that this difference is statistically significant, indicating that the observed variations in prevalence and mortality rates among the racial groups might be a result of factors that include racial disparities.

**Table 4:** ANOVA of prevalence and mortality among African Americans compared to other races with hypertension. NA, not applicable; SS, some of square; df, degree of freedom; MS, mean square; ANOVA, analysis of variance

	SS	df	MS	*F*-value	*P*-value
Between groups	1,680,536.50	3.00	560,178.83	118.22	0.001
Within groups	11,519,449.39	2,431	4,738.56	NA	NA
Total	13,199,985.89	2,434	NA	NA	NA

The within-group SS is 11,519,449.39 with 2,431 degrees of freedom, resulting in an MS of 4,738.56. This represents the variability within each racial group, accounting for individual differences and other factors not accounted for in the analysis. The total SS is 13,199,985.89, with 2,434 degrees of freedom, reflecting the overall variability in the prevalence and mortality rates among all the racial groups combined. These ANOVA results indicate that there are significant disparities in the prevalence and mortality rates related to hypertension among different racial groups. The higher *P*-value of <0.001 suggests that the observed differences in CVD outcomes among the racial groups are not due to random chance but are likely due to systematic differences. However, without the specific prevalence and mortality rate datasets, providing a more detailed interpretation of the magnitude of these disparities, including access to health, awareness levels, and economic status, is challenging.

Multiple comparisons of prevalence and mortality among African Americans compared to other races with hypertension

Table 5 shows the outcomes of multiple comparisons of prevalence and mortality rates among different racial groups with hypertension. The mean difference is representative of the variance in prevalence and mortality rates between the two racial groups being compared, while the standard error provides an estimate of the precision of the mean difference. The *P*-value indicates the statistical significance of the comparison, and the 95% CI gives the range within which the true mean difference is likely to fall.

**Table 5:** Multiple comparisons of prevalence and mortality between Black/African American, American Indian, Asian/Pacific Islander, and Whites. ^*^Mean difference is significant at 5% level. CI, confidence interval

Race		Mean difference	Standard error	95% CI	
				Lower bound	Upper bound
Black or African American	American Indian or Alaska Native	26.28526^*^	6.19	9.95	42.62
	Asian or Pacific Islander	47.20274^*^	4.71	34.77	59.64
	White	57.13789^*^	3.09	48.97	65.31

## Discussion

The study findings have indicated significant disparities in CVD prevalence and mortality rates between different racial groups in the United States. The findings answer the study objective, given that it highlights the higher burden of CVD and related conditions among African Americans compared to other racial groups. The study findings corroborate the findings of an earlier study, which disclosed that African American adults experienced a disproportionately higher prevalence rate of CVD and associated risk factors compared to their White counterparts in the United States [1].

Previous studies have reported that African Americans experience shorter life expectancies and higher mortality rates from CVDs, including coronary artery disease (CAD), hypertension, stroke, and obesity [2]. For this study, the ANOVA results, with a significant *F*-value and high *P*-value, suggest that the observed differences in CVD outcomes among racial groups are not random fluctuations but rather systematic disparities, including economic, racial, and structural disparities. These disparities likely stem from a complex interaction of genetic, biological, and social factors that disproportionately affect African Americans [12]. Despite advancements in healthcare and a decline in overall CVD mortality rates in the United States, Black Americans still face high CVD mortality rates.

Generally, hypertension plays a key role in the racial disparities in mortality rates between African Americans and Whites. It accounts for a substantial portion of the disparities and contributes to the increased risk of CVDs among African Americans [13]. The findings of the previous and present studies, therefore, highlight the urgent need for targeted interventions, prevention strategies, and healthcare initiatives that specifically address the higher prevalence and mortality rates of CVDs among African Americans. The ANOVA results emphasize the importance of considering racial disparities in cardiovascular health when formulating public health policies and interventions. Health promotion efforts should focus on addressing the social determinants of health, improving access to quality healthcare, promoting healthy lifestyle behaviors, and implementing culturally sensitive and tailored interventions for African American communities.

The interpretation of the results reveals important findings regarding the disparities in prevalence and mortality rates among different racial groups with hypertension. For instance, the comparison between African Americans and American Indian or Alaska Native individuals shows a significant difference in prevalence and mortality rates. African Americans have higher rates compared to American Indian or Alaska Native individuals, with a mean difference of -26.28526 (95% CI -42.62 to -9.95; *P* < 0.01). This suggests that African Americans face a greater burden of hypertension-related conditions and higher hypertension prevalence and mortality compared to American Indian or Alaska Native individuals.

Further, the comparison between African Americans and Asian or Pacific Islander individuals reveals a significant difference in prevalence and mortality rates. African Americans have significantly higher rates compared to Asian or Pacific Islander persons, with a mean variance of -47.20274 (95% CI -59.64 to -34.77; *P* < 0.01). The results, therefore, indicate that African Americans experience a greater burden of hypertension-related conditions compared to Asian or Pacific Islander individuals. However, in contradiction to the previous comparisons, the comparison between African Americans and Whites shows a significant variation in hypertension prevalence and mortality. Thus, African Americans have higher prevalence and mortality rates based on the *P*-value compared to White individuals, with a mean difference of 26.28526 (95% CI 9.95 to 42.62; *P* < 0.01). This finding is consistent with existing research, highlighting the racial disparities in cardiovascular health outcomes, where African Americans experience higher rates of hypertension-related conditions compared to Whites. For instance, recent studies have corroborated this study's findings by reporting that higher CVD prevalence and mortality rates are a significant challenge for African Americans and that nearly 30% of the deaths reported in the African American population can be attributed to hypertension [14]. In comparison to Whites, in Blacks, hypertension is more prevalent and tends to occur much earlier in life, in addition to being increasingly severe and normally linked to target organ injuries, including left ventricular hypertrophy and various cardiovascular complications [15].

Additionally, the comparisons between Asian or Pacific Islander and White individuals did not yield statistically significant differences in hypertension prevalence and mortality rates. The results, therefore, suggest that within the context of hypertension prevalence and mortality rates, these racial groups have comparable outcomes, indicating a similar burden of hypertension-related conditions. In general, the aforementioned findings corroborate the outcomes of various previous studies. For instance, the findings of the Third National Health and Nutrition Examination Survey (NHANES III) disclosed that Blacks have high age-adjusted and crude hypertension prevalence and mortality compared to Whites, Mexican Americans, and Natives [16]. Thus, the crude hypertension prevalence rates were 27.3% and 29.9% for Black women and men, respectively, in comparison to 23.8% and 25.6% for non-Hispanic women and men, as well as 14% and 14.6% for Mexican American women and men, concurrently. Thus, in Blacks, the overall age-adjusted hypertension prevalence was noted to be 32.4% [9].

Additionally, the findings of this study conform to those of initial research that indicates that despite the narrowing in the variations in prevalence and mortality rates between African Americans and Whites, the findings of initial studies indicated that African Americans were still twice more prone than Whites to die from CVD, which parallels the observed racial variations related to maternal health outcomes, for which CVD has been noted to be the main cause of mortality in the United States [4]. Moreover, socioeconomic status has been implied to be one of the fundamental causes of the key disease. Studies have disclosed that the objective measurement of socioeconomic status, including education level, subjective social standings, occupation, and income, were linked to hypertension, although there is not enough evidence indicating the correlation between socioeconomic status and hypertension development in Blacks [3,17]. Nevertheless, recent studies have disclosed the existence of associations between poverty and increased hypertension development risk among Blacks and Whites [8,17]. The notably higher premature CVD mortality rates among African Americans are considered a critically vital public health issue requiring urgent and concerted healthcare system, and federal, state, and community initiatives aimed at advancing the treatment and prevention of CVD risk factors and disease among African Americans [16].

Further, regarding the regional prevalence and mortality rate of hypertension among Blacks and other races, the findings have indicated that states with the highest hypertension mortality rates were Arizona, California, Texas, Florida, and Washington. On the contrary, states that include Vermont, North Dakota, and Wyoming had the lowest hypertension mortality and prevalence rates. Regionally, the northwest area had less mortality, with most of the mortality being concentrated in the southwestern and western states. The high prevalence in certain states might include the observation that Blacks have disproportionately higher severe hypertension rates, as it not only develops early in life but might be a result of their high propensity to develop obesity, which is one of the key risk factors for hypertension. Moreover, the high prevalence of Blacks in states such as Arizona, Washington, Florida, Texas, and California can be attributed to the large population of African Americans in the states compared to the others. The results of this study have provided additional evidence supporting the initial findings on regional disparities in the prevalence and mortality rates of cardiovascular disease. These disparities also highlight the contrasting gap between African American and White populations, which varies between urban and rural areas in the United States [1]. For instance, a previous study by Kyalwazi et al. [17] observed that the cardiovascular prevalence and mortality rates among African Americans were considerably higher in communities that had higher degrees of racial segregation in comparison to those communities/regions with low-to-moderate racial segregation levels [11,16]. Moreover, it was also noted that across the four census regions of the United States, African Americans consistently had higher age-adjusted cardiovascular prevalence and mortality rates compared to their White counterparts [17].

Study limitations

We utilized datasets from the CDC WONDER public files. The CDC WONDER datasets utilize ICD-10 coding in collection and storage. This may have several limitations that may have affected the outcome of our research. One limitation is that the accuracy of the recorded ICD-10 codes may vary. The coding process relies on accurate documentation by the healthcare provider of the patient's condition and assigning the appropriate code. Errors or inconsistencies in coding can occur, leading to incorrect classification or misrepresentation of the patient's actual condition. These coding inaccuracies can affect the reliability and validity of the data used for analyzing mortality rates. Another limitation is the potential lack of granularity in the ICD-10 codes for hypertension. The ICD-10 coding system provides a limited number of codes to classify different types and severity levels of hypertension. This lack of specificity may hinder the ability to differentiate between various subtypes of hypertension and their impact on mortality rates. Researchers might not be able to capture the nuances and distinctions necessary to accurately analyze mortality specifically related to hypertension. Furthermore, ICD-10 codes primarily focus on capturing diagnostic information rather than detailed clinical data or patient characteristics. While ICD-10 codes can provide an overview of the patient's condition, they may not capture important factors that could influence mortality, such as comorbidities, disease severity, treatment protocols, or other relevant clinical variables. These missing pieces of information may have limited the depth of analysis when assessing the relationship between hypertension and mortality. Finally, the other notable limitation is related to the potential biases. Thus, several potential biases might exist, including the response bias, which entails factors that vary from researchers’ perceived social status and appearance to the phrasing of the survey questions. For this study, potential response biases entail the observation that the cross-sectional study design used might preclude the assessment of the clinical outcomes associated with untreated and uncontrolled hypertension. Further, the other potential bias might be a result of researchers’ inability to effectively assess the divergences in hypertension cascade in different races and ethnic groups.

## Conclusions

During the study period (between 1999 and 2019), there was a significant increase in the prevalence of mortality due to hypertension among African Americans when compared to other races. These findings underscore the pressing need to address the increasing prevalence of hypertension and mortality rates among African Americans. Efforts should focus on addressing social determinants of health, improving access to quality healthcare, promoting healthy lifestyle behaviors, and implementing culturally sensitive interventions tailored for African American communities.
